# Fluorescence Lifetime Imaging of Human Sub-RPE Calcification In Vitro Following Chlortetracycline Infusion

**DOI:** 10.3390/ijms24076421

**Published:** 2023-03-29

**Authors:** Kavita R. Hegde, Adam C. Puche, Henryk Szmacinski, Kristina Fuller, Krishanu Ray, Nikita Patel, Imre Lengyel, Richard B. Thompson

**Affiliations:** 1 Department of Natural Sciences, Coppin State University, Baltimore, MD 21216, USA; 2 Department of Anatomy and Neurobiology, School of Medicine, University of Maryland, Baltimore, MD 21201, USA; 3 Department of Biochemistry and Molecular Biology, School of Medicine, University of Maryland, Baltimore, MD 21201, USA; 4 Institute for Human Virology, School of Medicine, University of Maryland, Baltimore, MD 21201, USA; 5 Department of Medicine, Renaissance School of Medicine at Stony Brook University, Stony Brook, NY 11794, USA; 6 The Wellcome-Wolfson Institute for Experimental Medicine, School of Medicine, Dentistry and Biomedical Science, Queen’s University Belfast, Belfast BT9 7BL, UK

**Keywords:** calcification, hydroxyapatite, retina, age-related macular degeneration, chlortetracycline, drusen, fluorescence lifetime microscopy, Alzheimer disease

## Abstract

We have shown that all sub-retinal pigment epithelial (sub-RPE) deposits examined contain calcium phosphate minerals: hydroxyapatite (HAP), whitlockite (Wht), or both. These typically take the form of ca. 1 μm diameter spherules or >10 μm nodules and appear to be involved in the development and progression of age-related macular degeneration (AMD). Thus, these minerals may serve as useful biomarkers the for early detection and monitoring of sub-RPE changes in AMD. We demonstrated that HAP deposits could be imaged in vitro by fluorescence lifetime imaging microscopy (FLIM) in flat-mounted retinas using legacy tetracycline antibiotics as selective sensors for HAP. As the contrast on a FLIM image is based on the difference in fluorescence lifetime and not intensity of the tetracycline-stained HAP, distinguishing tissue autofluorescence from the background is significantly improved. The focus of the present pilot study was to assess whether vascular perfusion of the well tolerated and characterized chlortetracycline (widely used as an orally bioavailable antibiotic) can fluorescently label retinal HAP using human cadavers. We found that the tetracycline delivered through the peripheral circulation can indeed selectively label sub-RPE deposits opening the possibility for its use for ophthalmic monitoring of a range of diseases in which deposit formation is reported, such as AMD and Alzheimer disease (AD).

## 1. Introduction

In the aging eye, lipid- and protein-containing sub-retinal pigment epithelial (sub-RPE) deposits, most often referred to as drusen, are often observed and are hallmarks of AMD, the most common cause of blindness in the elderly in developed countries [1] and have also been linked to other diseases (see the Discussion Section). Previously, we had shown that both hard and soft drusen, as well as basal linear deposits, exhibit calcification comprising spherules ranging upwards from 1 to 2 μm in diameter, up to 50+ micrometer nodules [2,3] composed largely of hydroxyapatite (Ca_10_(PO_4_)_6_(OH)_2_, HAP) or Whitlockite (Ca_9_(MgFe)(PO_4_)_6_PO_3_OH, Wht) but often with a lipid core. We proposed that these mineral deposits might nucleate the growth of drusen by selectively binding proteins typically found in drusen, such as vitronectin, amyloid beta, and complement factor H (CFH) [2,4]; variants of CFH are among the best-known risk factors for AMD [5]. The larger, less common nodules can be discerned by optical coherence tomography (OCT) as hyporeflective foci and represent a significant risk factor (odds ratio 6.4:1) for progression to advanced AMD (either geographic atrophy (GA) or choroidal neovascularization (CNV)) within one year [6]. However, the smaller spherules are difficult to detect by OCT even with current methods, such as adaptive optics, despite their relatively high refractive index. Taken together, these results suggested that HAP and Wht deposits might serve as a prognostic biomarker for the development of large and numerous mineral deposits and, ultimately, AMD, but that improved methods would be necessary for their detection. In addition, calcium mineral deposits in various forms are believed to either play a role or be an epiphenomenon in several other diseases, including Alzheimer Disease [7,8,9,10], as well as others. We note that the term “calcification” is broadly used to describe mineral deposits of many kinds, which frequently are forms of calcium phosphate, such as HAP or Wht, but it is sometimes the case that deposits described as “calcification” may be other calcium salts, and there are frequently not subjected to analysis to determine their chemical nature.

Initially, we identified the spherules in flat-mounted retina specimens in the microscope by use of tetracycline as a classic fluorescent stain that is selective for HAP [11] and tested other stains, such as Xylenol Orange and Alizarin Red S. Purpose-designed fluorescent labels for hydroxyapatite (e.g., the BoneTag family of stains (from LiCor) [12]) and the OsteoSense family [13] (Perkin-Elmer)) performed well in vitro with desirable long wavelength excitation and fluorescence emission, as well as better selectivity, but they have some drawbacks. In particular, while they have been used in animal studies for some years, their safety and pharmacokinetics in humans remain unknown; furthermore, the safety of strong illumination of the eye following administration of these cyanine dyes (cyanines were originally developed as photosensitizers for photography; reviewed in [14]) in animals or humans is unexplored. Moreover, a U.S. Food and Drug Administration-approved procedure for staining calcification in vivo in the retina with these compounds, especially the sub-RPE, remains to be developed. Particularly for a biomarker detection approach to be used as a screening tool, a simple, low-risk means of administration and detection are desirable.

Members of the tetracycline family of antibiotics classically known [11] to stain HAP also specifically stain sub-RPE HAP in the form of spherules and produce fluorescence emission in the greenish yellow portion of the spectrum [2]. While this fluorescence could be imaged on flat-mounted retinal tissue in the microscope, the substantial spectral overlap with the existing autofluorescence background of the retina [15] might compromise its sensitivity as a stain. Apart from this, tetracyclines have many attractive features: their overall safety in humans is well established, as well as their pharmacokinetic and ADME profiles [16,17]. Orally administered tetracyclines are known to stain teeth, particularly in children [11], suggesting the possibility of staining sub-RPE deposits through the bloodstream following oral administration. Some tetracyclines, such as minocycline and doxycycline, exhibit skin phototoxicity, which would be a potential concern in fluorescence ophthalmoscopic studies. However, there are no reports of phototoxicity for chlortetracycline (Aureomycin, or Cl-Tet) and its generally rapid clearance (hours) primarily through the kidneys suggests that waiting a period of hours following administration would lower circulating concentrations of the tetracycline and any consequent phototoxicity.

Our earlier studies and those of others suggest that particularly doxycycline and Cl-Tet might exhibit increases in apparent quantum yield upon binding authentic HAP. Such increases are often accompanied by increases in fluorescence lifetime, and we determined that this was the case: the average lifetime of Cl-Tet increased from 0.6 ns in neutral buffer to 1.7 ns bound to HAP, whereas doxycycline exhibited a more substantial increase from 0.6 to 3.8 ns. By use of fluorescence lifetime imaging microscopy (FLIM) [18] on post-mortem flat-mounted, Cl-Tet stained, aged human retinas we showed that HAP could indeed be imaged by its contrasting lifetime compared with the autofluorescence background of the retina [19]. Previously, the pioneering efforts of Schweitzer, Zinkernagel, Hammer, and their colleagues in developing fluorescence lifetime imaging ophthalmoscopy (FLIO) [20,21] demonstrated that under most circumstances the average autofluorescence lifetime of the retina in vivo is less than 0.4 ns, indicating that good lifetime contrast with HAP-bound Cl-Tet could also be expected in vivo.

Thus, we sought to test whether Cl-Tet in solution in the circulation would penetrate to label HAP in the sub-RPE space. We note that tetracyclines are known to penetrate the blood–brain barrier; however, this seemed unlikely to be an issue since the HAP deposits we sought to stain reside on the basal side of the RPE and are thus accessible to the choroidal circulation in any case. Using a cadaveric vascular infusion model, we tested whether circulating Cl-Tet would adequately label retinal HAP deposits so they could be distinguished by fluorescence lifetime imaging microscopy.

## 2. Results

**Figure 1:**
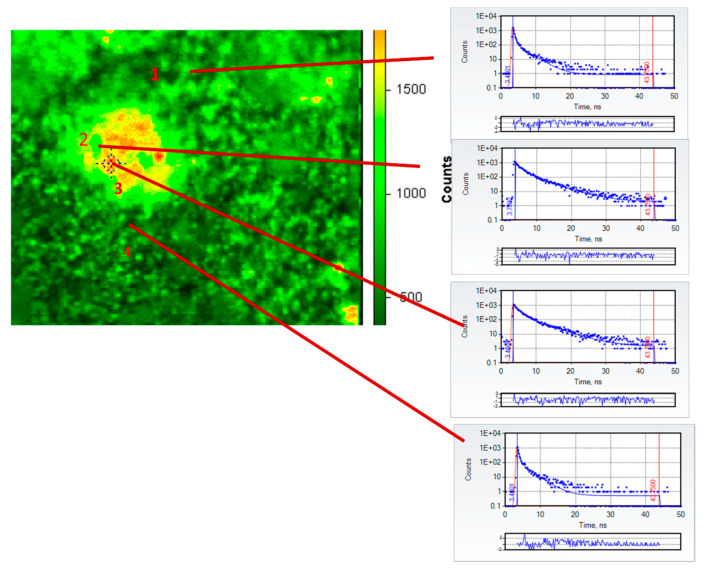
Fluorescence intensity micrograph (left) in false color of flat-mounted left retina of 82-year-old female (cause of death: congestive heart failure) following infusion with one liter of 500 mg/L Cl-Tet in PBS and a one-hour rinse with PBS. Excitation ≈442 nm (150 ps pulse width), emission 50 nm FWHM centered at 520 nm. Approximate size of the druse is 50 μm. Time domain fluorescence lifetime imaging microscopy (TD-FLIM) data were collected on an ISS ALBA FLIM through a 20 × 0.4 objective, and those of indicated regions of interest (1–4) in the image fit to two components (time decays to right) with the fitted parameters summarized in Table 1.

Following infusion with Cl-Tet and removal of the neurosensory retina from the flatmounted eye, we were able to image several clearly identifiable drusen under the fluorescence microscope. A fluorescence intensity micrograph in false color of a representative druse (approximately 50 μm in the longest dimension) of an 82-year-old female subject (cause of death: congestive heart failure) following infusion staining with chlortetracycline is shown in Figure 1 together with individual fluorescence decay curves of four selected regions of interest in the image: fields 2 and 3 are within the large druse and fields 1 and 4 are outside the druse in the retina. The TCSPC decays (blue dots) are fit by the standard nonlinear least squares procedure to two exponential decays; the red lines in the decays indicate the best fit; and the residuals (differences between the best fitted decay and the actual number of counts in each time bin, expressed in units of standard deviations) are plotted right underneath the decays. The derived values of lifetime τ*_i_* and preexponential factors α*_i_* for the ith component from the fits are summarized in Table 1.

**Figure 2:**
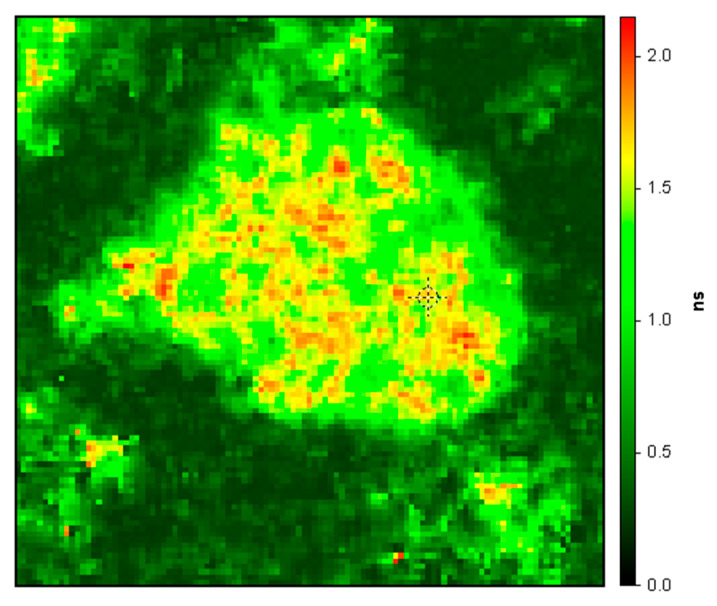
Close-up of druse in Figure 1 depicted in false color with the colors corresponding to the amplitude-weighted average fluorescence lifetimes (two component fit) of the individual pixels in nanoseconds (ns, vertical axis).

It is apparent from the decays themselves in Figure 1 and the derived fits that the decays from regions of interest within the druse are much longer than those outside the druse; this is also evident in Figure 2, which depicts in false color the amplitude-weighted average lifetime of the druse and its immediate surroundings. In particular, the average lifetimes of ROIs 2 and 3 in the druse were 1.35 and 1.49 ns, respectively, whereas the average lifetimes outside the druse in ROIs 1 and 4 were 0.36 and 0.34 ns, respectively. The two component fits are good as judged by the low reduced χ^2^ (all 1.0 ± 0.31) and the small, random residuals. The average lifetimes within the druse are similar to the lifetime measured previously (1.6 ns) for HAP-bound chlortetracycline [19]; those outside the druse are consistent with those measured previously (<0.4 ns) in the healthy retina by fluorescence lifetime imaging ophthalmoscopy [20,22]. We note that emission from outside the druse largely arises from the well-known pigments present particularly in the RPE, such as lipofuscin and melanolipofuscin, whose emission is substantially quenched as is evident from their short lifetimes. We recognize that the retinas in our experiments are not “healthy” per se as they derive from recently deceased donors, but the observed values match those from living subjects. It is also evident that the stained druse average lifetimes in Figure 2 are three to four-fold longer than those typically found in the retina under these spectral conditions, and thus by use of lifetimes the contrast is marked. It is unclear if the orange spots in the druse in Figure 2 indicate the presence of HAP spherules as we have previously shown; we suspect that in view of the known affinity of the tetracyclines for serum proteins, which strongly influence their pharmacokinetics, that they also may bind to the protein and lipid constituents of the drusen.

**Figure 3:**
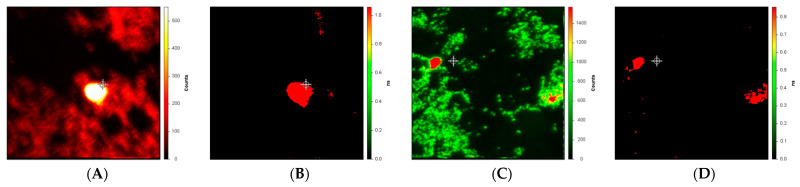
False color fluorescence intensity micrographs (**A**,**C**) and amplitude-weighted average fluorescence lifetime micrographs (**B**,**D**) of small drusen near the macula (**A**,**B**) and in the periphery (**C**,**D**) of the retina of a 79-year-old female (cause of death: dementia) stained by infusion with Cl-Tet. Conditions are as described in the legend to Figure 1 except that the background below less than 0.1% of peak intensity is not included in the lifetime images (**B**,**D**). Overall field size in all panels is 250 × 250 μm.

Figure 3 depicts intensity and lifetime images of drusen near the macula and in the periphery from a different subject, a 79-year-old female whose cause of death was listed as “dementia”. We presume that the cause of death was likely Alzheimer Disease, but this is not confirmed. The measurement and analysis of the lifetime images (Figure 3B,D) differ from those in Figure 2 only in that background intensity less than 0.1% of the peak is not included in the fits for the lifetime images. We note that while there is a substantial difference between the drusen lifetimes (roughly 1.0 nsec) and the background (roughly 0.1 nsec) in both the macula (Figure 3B) and periphery (Figure 3D), these numerical values are both smaller than we measured in Figure 1 (druse and background average lifetimes of roughly 1.4 and 0.4 ns, respectively).

## 3. Discussion

The principal finding of this work is that chlortetracycline can at least fluorescently label drusen and the calcification in the sub-RPE portion of the retina when infused through the bloodstream and permits their imaging based on contrast in fluorescence lifetimes. This is important because chlortetracycline can likely be administered orally to label the retinal calcification for imaging by fluorescence lifetime ophthalmoscopy, which as we have said may be a useful biomarker for early detection of AMD and other diseases. Our approach exploits the classic observation that tetracyclines are known to stain the teeth when administered orally [11]. While the eye is generally considered as part of the nervous system topologically, the location of this calcification beneath the retinal pigment epithelium (RPE) means it is exposed and accessible to the choroidal circulation beneath Bruch’s membrane and can thus be stained by agents in the bloodstream. Of course, many of the tetracyclines are administered orally; have robust safety data; have been in wide use for decades; and have known absorption, distribution, metabolism, and excretion (ADME) profiles; all of which makes them attractive when compared with other fluorescent stains of calcification, which have unknown ADME and pharmacokinetic profiles and toxicology in humans and which cannot be orally administered.

While we believe calcification in the retina may be a useful biomarker for predicting and following the course of AMD, calcified drusen in the retinal periphery has also been linked by ourselves and others to AD and even permit phenotyping the disease to an extent [7,8,9]. Most recently, we and our colleagues identified intracellular calcification in the brain as being linked to AD and to phosphorylated tau protein [10]. One of the subject’s cause of death was listed as “dementia”, and while this is not a firm diagnosis it is likely that this individual suffered from AD. We imaged some small drusen in the periphery as well as the macular region following infusion with Cl-Tet and identified regions of elevated intensity and lifetime similar to those observed in other subjects near the macula (Figure 3). Particularly, after suppressing low background intensity (below 0.1% of peak), the difference in amplitude-weighted average lifetime in the dementia patient between the drusen and background was very marked (Figure 3B,D) although the drusen average lifetime values themselves are evidently less than we had observed in the more central drusen (Figure 2). A larger study comparing the number, location, and size of such peripheral deposits observed by this method in Alzheimer patients with age-matched controls will be necessary.

While the Cl-Tet-stained druse in Figure 1 and Figure 2 and those we have previously observed [19] exhibit lifetimes close to that of what we measured previously for HAP-bound Cl-Tet, we hasten to add to what we pointed out above regarding the Cl-Tet potentially staining lipids and/or proteins in the drusen, and that it remains to be demonstrated how well Cl-Tet (or another tetracycline) can stain HAP in the retina before the druse has formed, and how successfully the HAP can be imaged alone in the retina prior to drusen formation. It furthermore remains to be demonstrated that HAP deposition in the sub-RPE is actually predictive of drusen formation and potentially diseases, such as AMD, and how well this procedure will work with FLIO in the living subject vs flat-mounted retinas. Experiments are underway to answer these questions.

## 4. Materials and Methods

A total of five recently deceased (mean post-mortem interval 18.6 h) elderly (mean age 80.6 years) donor cadavers were used in the study (summarized in Table 2). All donors were from the Maryland State Anatomy Board (SAB) with full donor informed consent and protocols approved by the SAB as well as the University of Maryland Baltimore Institutional Review Board (IRB). A total of 100–500 mg/L antibiotic (chlortetracycline hydrochloride, CAS No. [64-72-2]) (Amresco, Boise, ID, USA or Sigma, St. Louis, MO, USA) in isotonic saline was infused through the carotid artery over a period of approximately one hour at room temperature with a peristaltic pump simulating vascular pulsations using an open circulatory loop with the carotid infusate, allowed to drain out the jugular vein, followed by a rinse with PBS for a similar time. The doses were similar to human therapeutic doses of chlortetracycline, and while naturally cadaveric infusion does not exactly replicate the pharmacokinetics of orally administering the antibiotics, it does simulate oral administration. For some experiments, only one eye was stained so there would be a control, unstained eye from the same subject for comparison. In order to preferentially generate ocular perfusion through the vasculature we performed a modified carotid artery injection procedure. The stain was injected into the common carotid artery with the external carotid artery clamped directing peristaltic flow into the internal carotid artery and an outflow opening made in the external jugular vein. Since other arterial/venous branches have a static post-mortem fluid resistance from closed circulation, the targeted venous opening in the ipsilateral external jugular vein forms a preferential low resistance flowpath through the ophthalmic circulation. This pathway runs from the internal carotid artery into the ophthalmic artery and the retina vasculature with outflow through the inferior ophthalmic veins to the pterygoid venous plexus through the retromandibular and facial veins and then out the external jugular vein opening. The infusion experiment involved the following specific steps: (1) removal of the contralateral eye for control with clamping the proximal ophthalmic artery (i.e., proximal to the ethmoidal, retinal, or lacrimal branches). (2) Exposure of the common, external, and internal carotid arteries ipsilateral in the neck. (3) Insertion of the infusion canula into the common carotid artery with clamping of the external carotid artery. (4) Transection of the external jugular vein. (5) At the end of the infusion sequence, removal of the ipsilateral eye. Both eyes were enucleated, flatmounted, the neural retina removed, and the RPE/choroid complex examined by FLIM with the RPE on top, essentially as previously described [19], with further details in the figure legends.

## 5. Conclusions

We have shown that retinal hydroxyapatite can be stained in situ in *post mortem* eyes with chlortetracycline from within the circulation using simulated vascular concentration conditions and largely exhibits the expected lifetime behavior, indicating imaging in vivo with FLIO is likely feasible following oral administration of tetracycline.

## Figures and Tables

**Table 1:** Results of two-component fits of time-resolved data for selected ROIs of micrograph *.

ROI	τ_1_	α_1_	τ_2_	α_2_	<τ_α_>	χ^2^
1	0.36	0.97	2.88	0.03	0.44	1.01
2	1.35	0.93	5.28	0.07	1.62	1.10
3	1.49	0.95	5.81	0.05	1.72	1.31
4	0.34	0.97	2.66	0.03	0.41	0.77

* “ROI” refers to the regions of interest indicated in Figure 1; τ*_i_* and α*_i_* refer to the lifetime in nanoseconds and pre-exponential factor of the *i*th component, respectively, in the regions of interest in Figure 1; <τ_α_> is the amplitude-weighted average lifetime calculated for the region of interest; and χ^2^ is the reduced chi-squared for the best two-component fit for the region of interest.

**Table 2:** Donor Characteristics.

Age	Interval *	Gender	COD *
68	12	Female	Breast cancer
79	20	Female	Dementia
82	18	Female	Congestive heart failure
82	20	Female	End-stage renal disease
92	23	Male	Chronic obstructive pulmonary disease

* “Interval” is the approximate time interval in hours between death and infusion; “COD” is the cause of death assigned by the attending physician or coroner.

## Data Availability

Data will be made available to interested parties upon written request to the corresponding author.

## Abbreviations

AD: Alzheimer Disease; ADME: absorption, distribution, metabolism, and excretion; AMD: age-related macular degeneration; Cl-Tet, chlortetracycline; CNV: choroidal neovascularization; CFH: complement factor H; FLIM: fluorescence lifetime imaging microscopy; FLIO: fluorescence lifetime imaging ophthalmoscopy; GA: geographic atrophy; HAP: hydroxyapatite; OCT: optical coherence tomography; RPE: retinal pigment epithelium; TCSPC: time-correlated single photon counting; Wht: Whitlockite.
